# Imaging findings and clinical relevance of ^68^Ga-Pentixafor PET in atherosclerosis: a systematic review

**DOI:** 10.1186/s12880-023-01134-y

**Published:** 2023-10-26

**Authors:** Min Wang, Jiayu Zhang, Jiao Ma, Liyi Liu, Jia Wang, Chunyin Zhang

**Affiliations:** 1 Department of Nuclear Medicine, The Affiliated Hospital of Southwest Medical University, Luzhou, Sichuan PR China; 2 Nuclear Medicine and Molecular Imaging Key Laboratory of Sichuan Province, Luzhou, Sichuan PR China; 3 Academician (Expert) Workstation of Sichuan Province, Luzhou, Sichuan PR China; 4 Department of General Surgery (Breast Surgery), The Affiliated Hospital of Southwest Medical University, Luzhou, Sichuan PR China

**Keywords:** ^68^Ga-Pentixafor, Atherosclerosis, Imaging findings, Clinical relevance, A systematic review

## Abstract

**Objective:**

We aimed to perform a qualitative synthesis of evidence on the role of ^68^Ga-Pentixafor PET in atherosclerosis.

**Methods:**

A systematic search of the PubMed and Embase databases for studies reporting the evaluation of atherosclerotic lesions by ^68^Ga-Pentixafor PET was performed with a search time frame from database creation to 2022-12-26. The diagnostic test evaluation tool QUADAS-2 was used to evaluate the quality of the included literature and to perform descriptive analyses of relevant outcome indicators.

**Results:**

A total of 6 studies with 280 patients were included. One study reported only imaging outcome metrics, while the other five studies reported imaging outcome metrics and clinical correlation metrics. For imaging outcomes, three studies reported imaging results for ^68^Ga-Pentixafor PET only, and the other three studies reported imaging results for comparative analysis of ^68^Ga-Pentixafor PET with ^18^F-FDG PET. For clinical correlation, three studies reported the correlation between tracer uptake and cardiovascular risk factors, one study reported the correlation between tracer uptake and plaque calcification, and one study reported the correlation between all three: tracer uptake, cardiovascular risk factors, and plaque calcification.

**Conclusion:**

^68^Ga-Pentixafor PET has a good imaging effect on atherosclerotic lesions, and it is a promising imaging modality that may replace ^18^F-FDG PET for atherosclerosis imaging in the future. In patients with atherosclerosis, there is a clear clinical correlation between cardiovascular risk factors, tracer uptake, and plaque calcification.

## Introduction

Atherosclerosis is the pathological basis of cardiovascular disease. Unstable atherosclerotic plaque rupture, platelet aggregation and thrombosis lead to narrowing or occlusion of blood vessels, resulting in acute cardiovascular disease [1, 2], and it is one of the most common causes of death in the elderly. Because inflammation plays an important role in all stages of the atherosclerotic process [3], atherosclerosis is also considered to be a chronic inflammatory disease [4]. PET imaging can use biological processes to characterize high-risk features of atherosclerotic plaques that are prone to rupture. [^18^F]-fluorodeoxyglucose (^18^F-FDG) is the most commonly used radiotracer in vascular studies and can be used as a surrogate marker of plaque inflammation. However, the clinical application of ^18^F-FDG is somewhat limited. ^18^F-FDG can be taken up extensively by glucose-metabolizing cells. Structures such as the myocardium and neck can take up ^18^F-FDG in large amounts, which makes it difficult to accurately assess tracer uptake in the coronary arteries [5]. Therefore, the development of an alternative PET tracer with high specificity for arterial inflammation became necessary. Inflammatory cells overexpress the chemokine receptor type 4 (CXCR4), and ^68^Ga-Pentixafor is a novel PET tracer with high affinity and selectivity for CXCR4 [6]. Hyafil et al. reported ^68^Ga-Pentixafor a promising PET radiotracer that can be used to identify macrophage infiltration present in high-risk atherosclerotic plaques [7]. Therefore, we aimed to perform a qualitative synthesis of evidence on the role of ^68^Ga-Pentixafor PET in atherosclerosis.

## Materials and methods

The study strictly followed the PRISMA (Preferred Reporting Items for Systematic Reviews and Meta-analysis) guidelines, and the registration number on PROSPERO is CRD42023388079.

### Search strategy

PubMed and Embase databases were searched with a search time frame of build to 2022-12-26. Due to the small amount of published literature on Pentixafor, a single search term “Pentixafor” was used for a more comprehensive search of the literature related to ^68^Ga-Pentixafor PET assessment of atherosclerotic lesions. The literature on ^68^Ga-Pentixafor PET assessment of atherosclerotic lesions was then screened one by one. A manual supplemental search was also performed for all references in the included literature.

### Inclusion and exclusion criteria

#### Inclusion criteria

The literature was included in this study according to the principle of “PICOS”. (1) “Patients” with atherosclerosis; (2) ^68^Ga-Pentixafor PET as “intervention”; (3) ^18^F-FDG PET as a “comparator”; (4) Imaging results and clinical correlation as “outcomes” (Indicators of imaging results include site, amount, and the target-to-background ratios (TBR) of tracer uptake, agreement and correlation analysis of the two tracer uptakes); (5) Prospective or retrospective original research as “study type”.

#### Inclusion criteria

(1) Other types of publications, including conference abstracts, reviews, review articles, editorials and letters, etc.; (2) Articles with incomplete information and unable to extract valid data; (3) Literature with different research purposes; (4) Repeated publications.

### Literature screening and data extraction

Two investigators independently screened the literature in the order of title, abstract, and full text, and independently extracted basic information about the included literature, including first author, year of publication, country, study type, disease population, age, sample size, and outcome indicators, according to a pre-designed data extraction form. If relevant data were missing in the included literature, the corresponding authors were contacted by e-mail to obtain the data. When 2 investigators disagreed, this was resolved by discussion or consultation with the corresponding authors of this article.

### Quality evaluation

Two authors independently evaluated each study using the QUADAS-2 (Quality Assessment of Diagnostic Accuracy Studies) tool [8], and discrepancies were discussed and resolved by consensus with a third reviewer. The tool includes four domains: case selection, index testing, reference standard, process, and time. Each method was assessed according to the risk of bias, and the first three were also assessed according to questions of applicability. Each question is answered with “yes”, “no”, and “unclear”, and the degree of risk of bias can be judged as “low risk “, “high risk”, or “unclear risk”. Finally, the risk of bias for each included study was assessed using ReviewManager 5.4 software, and the risk of bias was plotted.

### Statistical processing

The database was created with Microsoft Excel 2021 software, entered in pairs, and proofread. When combining data, if 2 or more papers reported the same outcome indicator, Meta-analysis was performed using STATA17.0. The odds ratio (OR) and its 95% confidence interval (CI) were used for statistical data, and the mean difference (MD) and its 95% CI were used for measurement data. Conversely, only descriptive analyses of outcome indicators were performed when Meta-analysis was not feasible due to the reporting of outcome indicators in a single paper or heterogeneity among study populations.

## Results

### Literature screening results

**Fig. 1:**
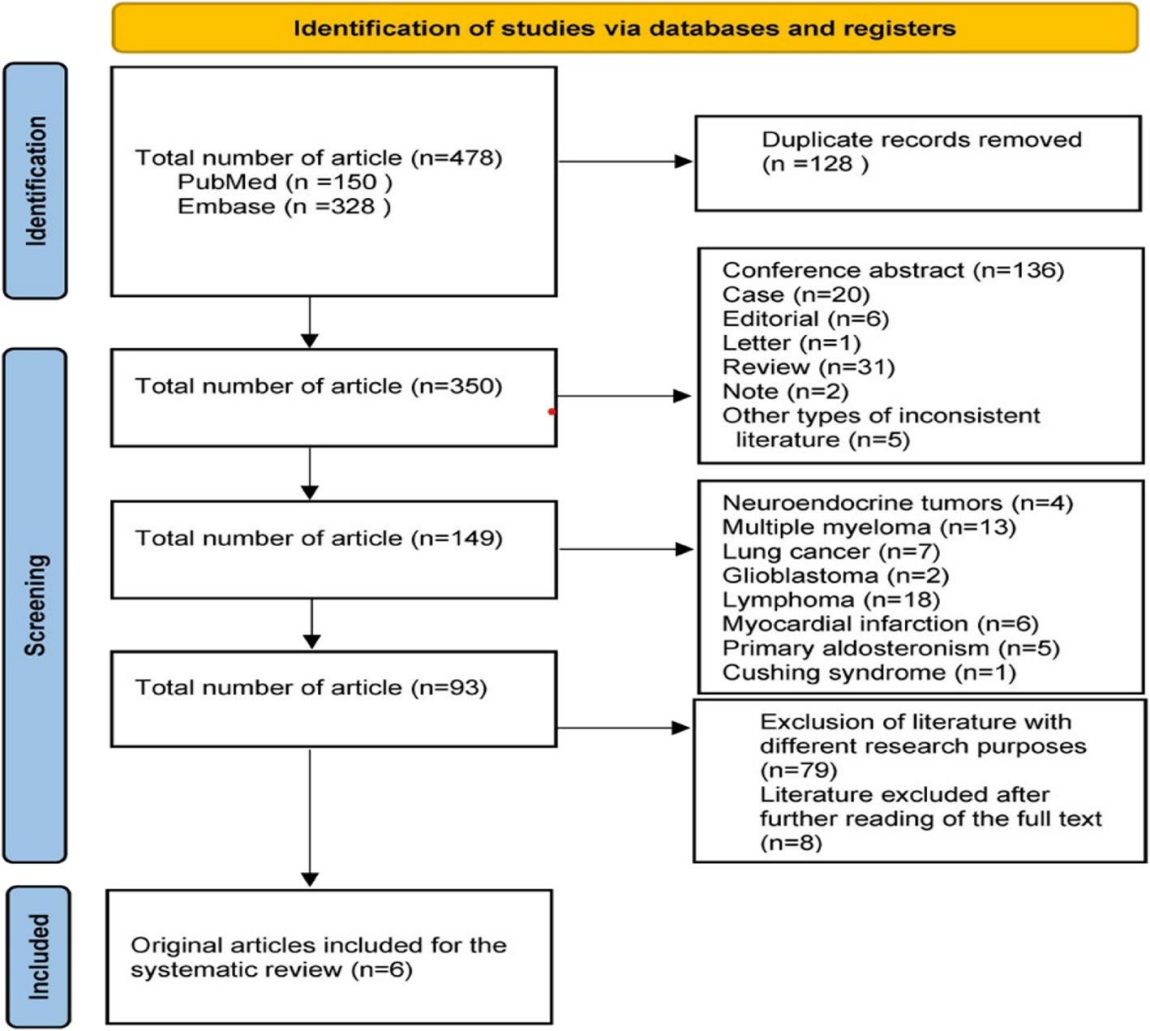
PRISMA flow chart included in the literature

The first 478 papers were detected, including 150 papers in PubMed and 328 papers in Embase. After reading the titles and abstracts, 201 publications were removed, including conference abstract (*n* = 136), case (*n* = 20), editorial (*n* = 6), letter (*n* = 1), review (*n* = 31), note (*n* = 2) and 5 other publications. Excluding 56 papers with incompatible study subjects, including neuroendocrine tumours (*n* = 4), multiple myeloma (*n* = 13), lung cancer (*n* = 7), glioblastoma (*n* = 2), lymphoma (*n* = 18), myocardial infarction (*n* = 6), primary aldosteronism (*n* = 5), and Cushing’s syndrome (*n* = 1). Excluding 79 papers with different study purposes and duplicate publications. Further reading of the full text excluded 8 papers without clinical outcomes and was unable to extract valid data. After the screening process, 6 studies were finally included [9–14]. The flow of the included literatures is shown in Fig. 1.

### Basic characteristics of included studies and quality evaluation results

Of the 6 included papers, 3 were prospective studies and 3 were retrospective studies. For the subject population, two studies were single subjects and four studies were non-single subjects. Subjects mainly include oncologic patients, patients with infection, etc. Cardiovascular risk factors included in the study included smoking, hypertension, dyslipidemia, diabetes, C-reactive protein (CRP) (≥ 3 mg/L), obesity, family history of cardiovascular disease and history of cardiovascular diseases. One study [10] reported only imaging outcome metrics and 5 studies [9, 11–14] reported imaging outcome metrics and clinical correlation metrics. Lawal et al. [10] reported imaging outcome indicators of atherosclerotic lesion uptake of ^68^Ga-Pentixafor and ^18^F-FDG; Lu et al. [9] reported imaging outcome indicators of lesion uptake of ^68^Ga-Pentixafor and ^18^F-FDG and correlation indicators of tracer uptake with cardiovascular risk factors; Kircher et al. [11] reported imaging outcome indicators of lesion uptake of ^68^Ga-Pentixafor and ^18^F-FDG and correlation indicators of tracer uptake with plaque calcification; Li et al. [12, 13] reported imaging outcome indicators of lesion uptake of ^68^Ga-Pentixafor and correlation indicators of tracer uptake with cardiovascular risk factors; Weiberg et al. [14] reported imaging outcome indicators of lesion uptake ^68^Ga-Pentixafor and also reported correlation indicators of tracer uptake with cardiovascular risk factors, correlation indicators of tracer uptake with plaque calcification and correlation indicators of cardiovascular risk factors with plaque calcification. The basic characteristics of the included studies are shown in Table 1.

**Table 1:** The basic characteristics of the included studies

Author	Year	The country of the subject	Prospective	Age	BMI (kg/m^2^)	Time interval	Subject population	CRF	Patients (M/F)	Imaging device
Lu [9]	2022	Austria a	N	68 ± 10	27.1	1 W	Lymphoma	Smoking, Hypertension, Dyslipidemia, Diabetes, CRP(≥ 3 mg/L)	19(11/8)	PET/ MRI
Lawal [10]	2020	SouthAfrica	Y	44.67 ± 7.62	24.18 ± 3.45	2 D	HIV-infected	Smoking, Hypertension, Diabetes, Family history of CVD	12(4/8)	PET/ CT
Kircher [11]	2020	Germany	N	62 ± 10	26	3 D	Multiple Myeloma Adrenocortical Cancer Neuroendocrine Tumour Non-Small Cell Lung Cancer Pleuramesothelioma Lymphoma Stomach Cancer Hepatocellulary-Carcinoma T-Cell Non-Hodgkin Lymphoma Small Cell Lung Cancer Pancreatic Cancer Thyroid Cancer Diffuse Large B-cell Lymphoma	Smoking, Hypertension, Diabetes, CRP(≥ 3 mg/L), Obesity, History of CVD	92(55/37)	PET/ CT
Li [12]	2019	Austria China	Y	61.8 ± 12.7	26.8 ± 4.0	NR	Mucosa-associated lymphoid tissue (MALT) lymphoma	Smoking, Hypertension, Dyslipidemia, Diabetes	72(45/27)	PET/ MRI
Li [13]	2018	NR	Y	67 ± 11	26 ± 4	3 W	Lymphoma Pancreatic cancer	Smoking, Hypertension, Dyslipidemia, Diabetes, Family history of CVD, History of CVD	34(17/17)	PET/ MRI
Weiberg [14]	2018	Germany	N	59.5 ± 16.2	NR	NR	Interstitial lung disease Sarcoidosis Complicated urinary tract infection Leukemia Miscellaneous	Smoking, Hypertension, Dyslipidemia, Diabetes, History of CVD	51(39/12)	PET/ CT

*N* no, *Y* yes, *D* day, *W* week, *M* male, *F* female, *CRF* Cardiovascular Risk Factors, *CVD* Cardiovascular Disease, *CRP* C−reactive protein, *HIV* Human immunodeficiency virus

**Fig. 2:**
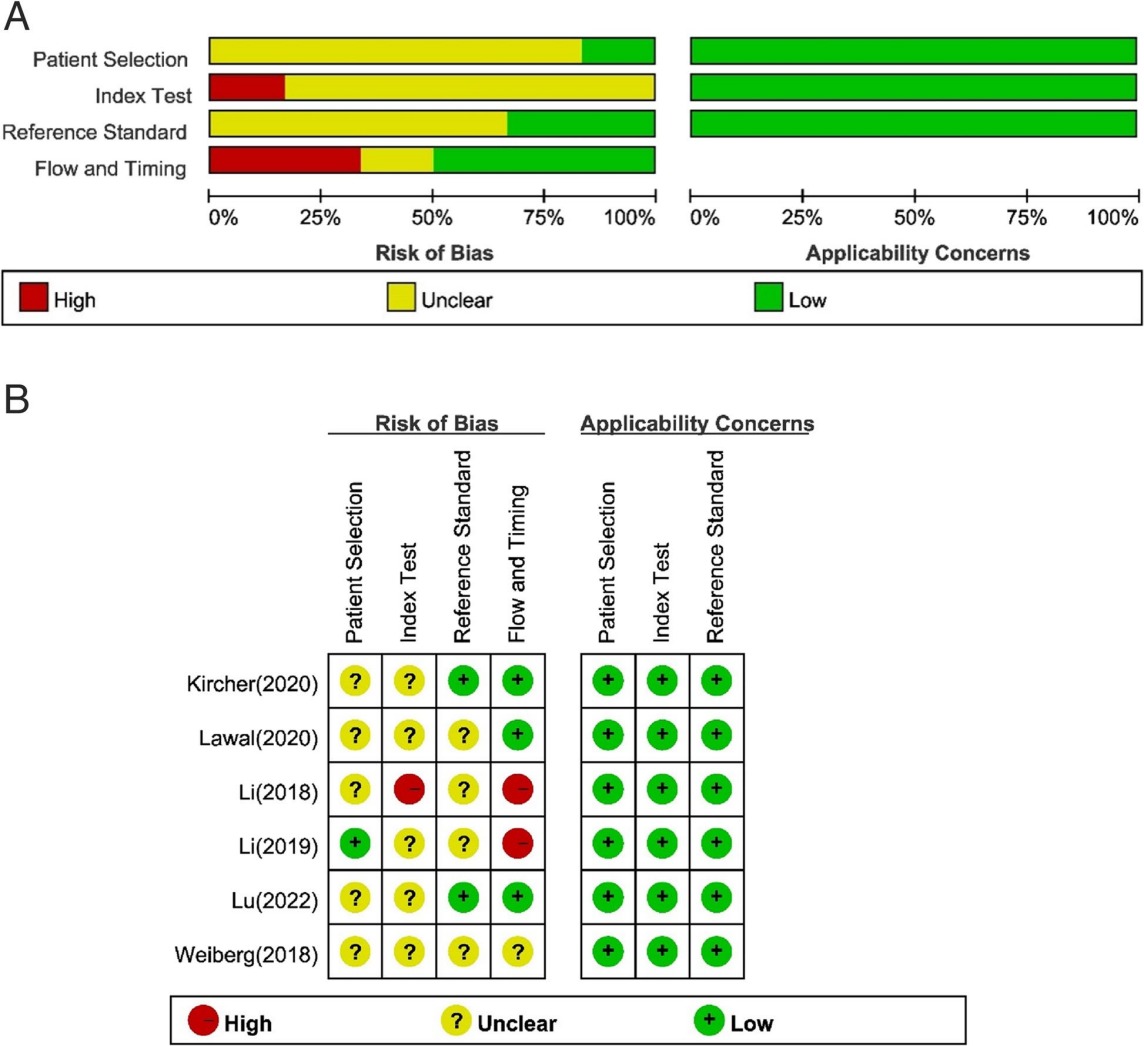
**A** Quality evaluation results of included documents. **B** Quality evaluation results of included documents

 Quality evaluation was performed with the QUADAS-2 tool. Risk of bias: for case selection, 5 studies [9–11, 13, 14] were medium risk, with the main risk arising from continuity or randomization of patient inclusion; for trials to be evaluated, 1 study [13] was high risk and 5 studies [9–12, 14] were medium risk, with the main risk arising from the implementation of blinding and the determination of thresholds; for gold standard 4 studies [10, 12–14] were medium risk, with the main risk arising from the implementation of the blinding method; for case, flow and progression, 2 studies [12, 13] were high risk and 1 study [14] was a medium risk, with the main risk arising from the completeness of the case inclusion analysis and the appropriate interval. All studies had a low risk of clinical applicability. The quality assessment of the included literature is shown in Fig. 2 (a) (b).

### Systematic evaluation results

Limited by clinical heterogeneity with different reported outcome indicators, among other reasons, only descriptive analysis was performed in this study. (The comparison between ^68^Ga-Pentixafor and ^18^F-FDG is presented in Table 2. The abstracts of the included literatures are shown in Table 3).

**Table 2:** Analysis of imaging results of tracer uptake in atherosclerotic lesions

Author	^68^Ga -Pentixafor PET	^18^ F-FDG PET
Lu [9]	lesion-based analysis: number 88%,TBR 1.9 patient-based analysis TBR 1.85 ± 0.20	lesion-based analysis: number 48%,TBR 1.63 ± 0.29 patient-based analysis TBR 1.42 ± 0.19
Lawal [10]	NR	early aorta: TBR 1.76 ± 0.3 late aorta: TBR 2.76 ± 0.52 early carotid artery: TBR 1.51 ± 0.38 late carotid artery: TBR 2.38 ± 0.66
Kircher [11]	lesion-based analysis: TBR 1.8 ± 0.5 patient-based analysis number 4(0–13), TBR 1.8 ± 0.30	lesion-based analysis: TBR 1.4 ± 0.4 patient-based analysis number 1(0–10), TBR 1.4 ± 0.30
Li [12]	group 1: TBRmax 1.29 ± 0.21 group 2: TBRmax 1.57 ± 0.27 group 3: TBRmax 1.64 ± 0.37 group 4: TBRmax 1.55 ± 0.26	NR
Li [13]	descending aorta: number 225, TBRmax 1.9 ± 0.4 abdominal aorta: number 168, TBRmax 1.9 ± 0.4 aortic arch: number 83, TBRmax 1.8 ± 0.2 common carotid artery: number 74, TBRmax 1.7 ± 0.3 ascending aorta: number 61, TBRmax 1.7 ± 0.2	NR
Weiberg [14]	right common carotid artery: number 49, TBR 1.7 ± 0.4 left common carotid artery: number 55, TBR 1.6 ± 0.4 thoracic aorta: number 339, TBR 1.9 ± 0.4 abdominal aorta: number 369, TBR 2.1 ± 0.6 right iliac artery: number 115, TBR 1.9 ± 0.4 left iliac artery: number 115, TBR 2.0 ± 0.5 right femoral artery: number 180, TBR 1.9 ± 0.5 left femoral artery: number 189, TBR 2.1 ± 0.6	NR

**Table 3:** Abstracts of included documents

Author	Abstract
Lu [9]	Objective: This study compared ^68^Ga-Pentixafor uptake in active arterial segments with corresponding ^18^F-FDG arterial uptake as well as the relationship with cardiac ^68^Ga-Pentixafor uptake. Conclusion: ^68^Ga-Pentixafor PET/MRI identified many more lesions than ^18^F-FDG PET/MRI. Patients with high-risk cardiovascular factors illustrated an increased uptake of ^68^Ga-Pentixafor. There was a correlation between the elevated uptake of ^68^Ga-Pentixafor in the active arterial segments and heart.
Lawal [10]	Objective: In this study we aimed to perform a head-to-head comparison of ^18^F-FDG PET/CT and ^68^Ga-Pentixafor PET/CT for quantification of arterial inflammation in PLHIV. Conclusion: We found a high level of agreement in the quantification variables obtained using ^18^F-FDG PET and ^68^Ga-Pentixafor PET. There is a good level of agreement in the arterial tracer quantification variables obtained using ^18^F-FDG PET/CT and ^68^Ga-Pentixafor PET/CT in PLHIV. This suggests that ^68^Ga-Pentixafor may be applied in the place of ^18^F-FDG PET/CT for the quantification of arterial inflammation.
Kircher [11]	Objective: The aim of this retrospective study was to investigate the performance of ^68^Ga-Pentixafor PET/CT for imaging atherosclerosis in comparison to ^18^F-FDG PET/CT. Conclusion: CXCR4-directed imaging of the arterial wall with ^68^Ga-Pentixafor PET/CT identified more lesions than ^18^F-FDG PET/CT, with only a weak correlation between tracers.
Li [12]	Objective: We aimed to evaluate ^68^Ga-Pentixafor PET in combination MRI for in vivo quantification of CXCR4 expression in carotid plaques. Conclusions: In vivo evaluation of CXCR4 expression in carotid atherosclerotic lesions is feasible using ^68^Ga-Pentixafor PET/MRI. In atherosclerotic plaque tissue, CXCR4 expression might be used as a surrogate marker for inflammatory atherosclerosis.
Li [13]	Objective: We sought to evaluate human atherosclerotic lesions using ^68^Ga-Pentixafor PET/MRI. Conclusion: Patients with high arterial uptake showed increased incidence of cardiovascular risk factors, suggesting apotential role of ^68^Ga-Pentixafor in characterization of atherosclerosis.
Weiberg [14]	Objective: The aim of this study was to assess the prevalence, pattern, and clinical correlates of arterial wall accumulation of ^68^Ga-Pentixafor, a specific CXCR4 ligand for PET. Conclusion: ^68^Ga-Pentixafor PET/CT is suitable for non-invasive, highly specific PET imaging of CXCR4 expression in the atherosclerotic arterial wall. Arterial wall ^68^Ga-Pentixafor uptake is significantly associated with surrogate markers of atherosclerosis, and is linked to the presence of cardiovascular risk factors. ^68^Ga-Pentixafor signal is higher in patients with a high-risk profile, and may hold promise for identification of vulnerable plaque.

*PLHIV* People living with human immunodeficiency virus, *CXCR4* Chemokine receptor type 4

#### Analysis of imaging results of tracer uptake in atherosclerotic lesions

Lu et al. [9] retrospectively analyzed 19 patients with lymphoma, and in a lesion-based analysis, ^68^Ga-Pentixafor PET detected more lesions than ^18^F-FDG PET (88% vs. 48%, *p* < 0.001) and showed higher uptake than ^18^F-FDG PET (TBR: 1.90 ± vs. 1.63 ± 0.29, *p* < 0.001); ^68^Ga-Pentixafor uptake was also significantly higher than ^18^F-FDG in patient-based analysis (TBR: 1.85 ± 0.20 vs. 1.42 ± 0.19, *p* < 0.001). Lawal et al. [10] prospectively included 12 AIDS patients and performed ^68^Ga-Pentixafor PET and ^18^F-FDG PET imaging of the patients. For analysis of ^18^F-FDG PET imaging, TBR was elevated and statistically significant on delayed scans of both aorta (early: 1.76 ± 0.3, delayed: 2.76 ± 0.52, t: -5.738, *p* < 0.001) and carotid artery (early: 1.51 ± 0.38, delayed: 2.38 ± 0.66, t: -4.741, *p* = 0.001). significance. Correlation analysis of the two imaging modalities showed a positive correlation between the TBR of the early aorta (*r* = 0.344, *p* = 0.274), late aorta (*r* = 0.225,*p* = 0.483), early carotid artery (*r* = 0.123, *p* = 0.704) and late carotid artery (*r* = 0.295, *p* = 0.352), but neither reached statistical significance. Analysis of the agreement between the two imaging modalities showed good agreement between the two imaging modalities, and the degree of agreement was higher for early scans than for delayed scans. Kircher et al. [11] retrospectively analyzed a total of 652 lesions detected in 92 patients, and for each patient, the median number of positive lesions was 4 (0–13) for ^68^Ga-Pentixafor PET compared to 1 (0–10) for ^18^F-FDG PET, and the number of positive lesions for ^68^Ga-Pentixafor PET correlated moderately with ^18^F-FDG PET-positive lesion (*r* = 0.46, *P* < 0.0001); the mean TBR of ^68^Ga-Pentixafor PET was significantly higher than that of ^18^F-FDG PET (1.8 ± 0.5 vs. 1.4 ± 0.4, *P* < 0.01), and the ^68^Ga-Pentixafor PET and ^18^F-FDG PET TBR showed a weak positive correlation; based on patient analysis, individual mean TBR was significantly higher for ^68^Ga-Pentixafor PET than for ^18^F-FDG PET (1.8 ± 0.3 vs. 1.4 ± 0.3, *P* < 0.001 ), and there was a modest correlation (*r* = 0.36, *P* < 0.001). Li et al. [12] analyzed 72 patients and grouped them, showing that patients in group 1 (non-eccentric carotid atherosclerotic lesions, *n* = 27, TBRmax = 1.29 ± 0.21) had significantly lower ^68^Ga-Pentixafor uptake than those in group 2 (mild eccentric carotid atherosclerotic lesions, *n* = 67, TBRmax = 1.57 ± 0.27), group 3 (moderate eccentric atherosclerotic carotid lesions, *n* = 41, TBRmax = 1.64 ± 0.37) and group 4 (severe eccentric atherosclerotic carotid lesions, *n* = 19, TBRmax = 1.55 ± 0.26) (*p* < 0.05), whereas between groups 2, 3 and 4 ^68^Ga-Pentixafor uptake were not statistically different. Li et al. [13] included 34 patients in the study and ^68^Ga-Pentixafor PET detected a total of 611 (TBRmax = 1.8 ± 0.4) lesions, with the descending aorta being the vessel segment with the highest number of lesions and strongest tracer uptake (*n* = 225, TBRmax = 1.9 ± 0.4), followed by the abdominal aorta (*n* = 168, TBRmax = 1.9 ± 0.4), aortic arch (*n* = 83, TBRmax = 1.8 ± 0.2), common carotid artery (*n* = 74, TBRmax = 1.7 ± 0.3) and ascending aorta (*n* = 61, TBRmax = 1.7 ± 0.2). Weiberg et al. [14] retrospectively analyzed a total of 1411 (TBR = 2.0 ± 0.5) lesions in 51 patients with the following uptake characteristics: right common carotid artery (*n* = 49, TBR = 1.7 ± 0.4), left common carotid artery (*n* = 55, TBR = 1.6 ± 0.4), thoracic aorta (*n* = 339, TBR = 1.9 ± 0.4), abdominal aorta (*n* = 369, TBR = 2.1 ± 0.6), right iliac artery (*n* = 115, TBR = 1.9 ± 0.4), left iliac artery (*n* = 115, TBR = 2.0 ± 0.5), right femoral artery (*n* = 180, TBR = 1.9 ± 0.5), and left femoral artery (*n* = 189, TBR = 2.1 ± 0.6).

#### Clinical correlation analysis of tracer uptake in atherosclerotic lesions

Lu et al. [9] retrospectively analyzed the relationship between tracer uptake and cardiovascular risk factors in 19 patients with lymphoma and showed that comparing the high-risk group (*n* = 9) with cardiovascular risk factors to the low-risk group (*n* = 10), TBR was significantly increased in active lesions of ^68^Ga-Pentixafor (2.02 ± 0.15 vs. 1.86 ± 0.10, *p* = 0.015), but this was not found for ^18^F-FDG (1.85 ± 0.10 vs. 1.80 ± 0.07, *p* = 0.149). Kircher et al. [11] analyzed the relationship between tracer uptake and plaque calcification in 92 patients and found an inverse relationship between the degree of plaque calcification and the intensity of uptake of both tracers (as measured by TBR), with non-calcified lesions (*n* = 467) showing the highest TBR values for both tracers (1.9 ± 0.4 and 1.5 ± 0.4, respectively), mildly calcified lesions (*n* = 99) showed higher TBR values for both (1.7 ± 0.4 and 1.3 ± 0.3, respectively, *P* < 0.01), while severely calcified lesions (*n* = 86) showed the lowest TBR values (1.4 ± 0.6 and 1.1 ± 0.4, respectively). TBR was higher in ^68^Ga-Pentixafor PET than in ^18^F-FDG PET when analyzing different subgroups of calcification. Li et al. [12] analyzed 72 patients and found a significant correlation between ^68^Ga-Pentixafor uptake (TBRmax) and the prevalence of hypertension (Pearson’s *r* = 0.27/ Pearson’s r *r* = 0.35, *p* < 0.05), and there was a significant correlation between the prevalence of type II diabetes mellitus (Pearson’s *r* = 0.27/ Pearson’s r *r* = 0.35, *p* < 0.05). Li et al. [13] analyzed the correlation between tracer intake and cardiovascular risk factors. The results showed that in patients with TBR > 1.7, patients with diabetes, hypercholesterolemia, and cardiovascular history accounted for 27.3%, 36.4%, and 36.4% respectively, while in patients with TBR ≤ 1.7, patients with diabetes, hypercholesterolemia and cardiovascular history only accounted for 0%, 8.3%, and 8.3%. (*P* < 0.05) This shows that when TBR > 1.7, the high-risk group of cardiovascular risk factors is more likely to appear. At the same time, by comparing and analyzing the TBR values of patients in a high-risk group and a low-risk group of cardiovascular risk factors, the results showed that the TBR values in the high-risk group were significantly higher than those in the low-risk group (1.9 ± 0.3 vs. 1.7 ± 0.2, *p* < 0.05). Weiberg et al. [14] retrospectively analyzed 51 patients and found significant correlations between the number of cardiovascular risk factors and the number of calcified plaques (*r* = 0.46, *P* = 0.0007), the number of lesions with tracer ingestion (*r* = 0.70, *P* < 0.0001) and TBR (*r* = 0.36, *P* = 0.009). Univariate regression analysis showed significant correlations between the number of lesions for tracer uptake and age at risk (*r* = 0.60, *P* < 0.0001), arterial hypertension (*r* = 0.56, *P* < 0.0001), hypercholesterolemia (*r* = 0.47, *P* = 0.0005), smoking history (*r* = 0.35, *P* = 0.01), and previous vascular events (*r* = 0.47, *P* = 0.0004); multiple regression analysis showed that age at risk (*r* = 0.50, *P* = 0.0003), arterial hypertension (*r* = 0.52, *P* = 0.0001), and smoking history (*r* = 0.36, *P* = 0.01) were all independently associated with atherosclerotic lesions. There was a statistically significant association between the number of lesions ingested with tracer and calcified plaque burden (*r* = 0.67, *P* < 0.0001), maximum plaque thickness (*r* = 0.56, *P* < 0.0001), and calcification score (*r* = 0.69, *P* < 0.0001), all of which described different aspects of the degree of arterial calcification. Also, there was a significant correlation between calcified plaque burden and age at risk (*r* = 0.51, *P* = 0.0001), arterial hypertension (*r* = 0.37, *P* = 0.008), and prior vascular events (*r* = 0.46, *P* = 0.0008); multiple regression analysis showed that calcified plaque burden was associated with age at risk (*r* = 0.49, *P* = 0.0003) and prior vascular events (*r* = 0.38, *P* = 0.008) were independently associated.

## Discussion

Atherosclerosis is a chronic systemic disease in which inflammation is a dynamic trigger for progression [15–17]. Progressive systemic enlargement of atherosclerotic plaques leads to a range of debilitating cardiovascular diseases, including peripheral arterial disease, ischemic stroke, coronary artery disease, and acute myocardial infarction [18]. These diseases are the leading cause of morbidity and mortality in the United States and worldwide [19–21]. Conventional imaging examinations (including ultrasound, CT, and MRI angiography) have limited ability to assess the early stages of atherosclerosis [22, 23]. Therefore, molecular imaging offers an attractive opportunity to examine the pathological features of atherosclerotic disease at the microscopic level [24]. Studies have suggested that ^68^Ga-Pentixafor may be a potential imaging molecule for atherosclerosis, but the use of ^68^Ga-Pentixafor PET for imaging atherosclerotic lesions is not yet widely used in clinical practice due to the lack of evidence-based medical evidence.

To further clarify the role of ^68^Ga-Pentixafor PET in atherosclerotic, we aimed to perform a qualitative synthesis of evidence on the role of ^68^Ga-Pentixafor PET in atherosclerosis for the first time. The results of this study found that ^68^Ga-Pentixafor PET has better imaging results than ^18^F-FDG PET and can overcome some limitations of ^18^F-FDG PET imaging, and may be able to replace ^18^F-FDG PET for atherosclerosis imaging in the future. In addition, there is a clear clinical correlation between cardiovascular risk factors, tracer uptake and plaque calcification.

Specifically, a comparative analysis of imaging results between ^68^Ga-Pentixafor PET and ^18^F-FDG PET revealed good agreement and correlation between the two imaging modalities, and higher uptake of tracers (both in terms of quantity and intensity of uptake) by patients and lesions with ^68^Ga-Pentixafor PET compared to ^18^F-FDG PET. In addition, it was found that the uptake of tracers was higher in eccentric carotid atherosclerotic lesions than in non-eccentric carotid atherosclerotic lesions. In terms of clinical correlation, the uptake of tracers (both quantity and TBR) by patients and lesions increased with the number of cardiovascular risk factors in patients, the number of plaque calcifications increased with the number of cardiovascular risk factors, and the uptake of tracers by lesions decreased instead with the increased burden of plaque calcification, suggesting a positive correlation between cardiovascular risk factors and tracer uptake and plaque calcification; whereas a negative correlation existed between tracer uptake and the degree of plaque calcification.

Atherosclerosis is a global health problem. Although some progress has been made in understanding the complex underlying biology of atherosclerosis, we still need radioactive tracers targeting molecular changes in vulnerable plaques to identify vulnerable plaques and prevent adverse events. At present, the diagnostic localization of ^68^Ga-Pentixafor still needs to be improved. Bartlett et al. [25] summarized the efficacy of various radioactive tracers for PET imaging in plaque characterization and risk assessment. This study thought that further elucidating the potential biological mechanism of CXCR4 would help to improve the understanding of the clinical application of this radioactive tracer. In addition, an accurate estimate of the tracer uptake in vascular lesions is extremely challenging given the small size of the lesions compared to the spatial resolution of PET. Some studies [26, 27] have shown that vascular inflammation imaging with ^18^F-FDG PET requires optimized imaging conditions. The research results of Lawal et al. [28] have shown that vascular quantification can be improved by increasing the uptake of vascular tracers and improving the clearance of blood-pool background activity. Therefore, a set of standard imaging protocols and quantitative methods is very important for molecular imaging of vascular inflammation.Some limitations remain in this study. First, given the relative novelty of the Pentixafor tracer, only a few studies (*n* = 6) were available for review. Some of them were conducted by the same study group and possible overlap of patient data cannot be excluded exclusively based on the information reported in the manuscript. In addition, most studies included a different population of subjects. The TBR values defining positive atherosclerotic lesions in the included studies were not all the same. All of the above factors could be sources of heterogeneity in this study. Due to the limitations of clinical heterogeneity and different outcome indicators, only a descriptive analysis was performed in this study, and a large number of prospective randomized studies are needed in the future to further validate the clinical utility of ^68^Ga-Pentixafor PET application in atherosclerosis.

## Conclusion

In this study, a systematic evaluation of ^68^Ga-Pentixafor PET for atherosclerosis imaging was performed. The results showed that ^68^Ga-Pentixafor PET has a good imaging effect on atherosclerotic lesions, and it is a promising imaging modality that may replace ^18^F-FDG PET for atherosclerosis imaging in the future. In patients with atherosclerosis, there is a clear clinical correlation between cardiovascular risk factors, tracer uptake, and plaque calcification.

## Data Availability

The dataset(s) supporting the conclusions of this article is(are) included within the article.

## Abbreviations

^18^F-FDG: [^18^F]-fluorodeoxyglucose
CXCR4: Chemokine receptor type 4
TBR: Target-to-background ratios
OR: Odds ratio
CI: Confidence interval
MD: Mean difference
AIDS: Acquired immune deficiency syndrome
CRP: C-reactive protein
